# Post-mastectomy pain syndrome and sleep disorders: effects of physical activity

**DOI:** 10.3389/fspor.2025.1579635

**Published:** 2025-11-20

**Authors:** Marco Calapai, Luisa Puzzo, Giuseppe Bova, Daniele Alfio Vecchio, Rosario Blandino, Alessia Barbagallo, Ilaria Ammendolia, Luigi Cardia, Filippo Firenzuoli, Fabrizio Calapai, Mariaconcetta Currò, Giovanni Ficarra, Emanuela Esposito, Fabio Trimarchi, Debora Di Mauro, Gioacchino Calapai, Carmen Mannucci

**Affiliations:** 1 Breast Unit, San Vincenzo Hospital, Azienda Sanitaria Provinciale Messina, Messina, Italy; 2 Pain Therapy Unit, San Vincenzo Hospital, Azienda Sanitaria Provinciale Messina, Messina, Italy; 3 Department of Chemical, Biological, Pharmacological and Environmental Sciences, University of Messina, Messina, Italy; 4 Department of Human Pathology of Adult and Childhood “Gaetano Barresi”, University of Messina, Messina, Italy; 5 School of Human Health Sciences, School of Specialization in Anesthesia, Intensive Care and Pain Medicine, University of Florence, Florence, Italy; 6 Department of Biomedical and Dental Sciences and Morphological and Functional Imaging, University of Messina, Messina, Italy; 7 Department of Clinical and Experimental Medicine, University of Messina, Messina, Italy; 8 Genetics and Pharmacogenetics Unit, Policlinico Universitario “G. Martino”, University of Messina, Messina, Italy

**Keywords:** sleep disorders, post-mastectomy pain syndrome, chronic pain, physical activity, breast cancer

## Abstract

**Introduction:**

Aim of the study was the evaluation of physical activity effects on chronic post-surgery pain perception, sleep quality and related biomarkers (melatonin, AdrenoCorticoTropic Hormone, and cortisol) in women undergoing postmastectomy.

**Methods:**

A prospective observational unicentric cohort study was designed by recruiting women undergoing unilateral or bilateral mastectomy. One hundred and eighty (180) women were enrolled in the study according to inclusion and exclusion criteria. The mean age of the sample of patients recruited for the study was 50.34 ± 11.9 years (range 28–72 years, median age 53.5 years). The mean BMI was 21.59 ± 1.49. All the participants were Caucasian. Pain assessment, quality of sleep and motor activity of each participant in the study was measured 3 and 6 months after the intervention as well as biomarkers.

**Results:**

show that level of physical activity measured through International Physical Activity Questionnaire reduces intensity of pain and its interference with quality of life, moreover, in women with higher physical activity, results show a reduction of sleep disturbance, cortisol and ACTH levels and an increase of melatonin, compared with women with minor motor activity and lower physical activity level.

**Conclusions:**

Physical activity seems to reduce pain intensity, reduce sleep disorders together with a reduction of cortisol and ACTH and an increase of melatonin. (Clinical Trial.gov identifier: NCT06803563)

## Introduction

Following surgery, chronicization of pain is common, which can result in psychiatric issues and functional limits that negatively affect the quality of life (1). Chronic post-surgical pain (CPSP) has been defined as “Pain that develops after surgical intervention and lasts at least 2 months, other causes of pain have to be excluded, in particular, pain from a condition existing before the surgery” (2). “Pain persisting at least three months after surgery, that was not present before surgery, or that had different characteristics or increased intensity from preoperative pain, localized to the surgical site or a referred area, and other possible causes of the pain were excluded (e.g., cancer recurrence, infection)” is an updated definition of CPSP (3, 4).

In the 1970s, as a part of a study about a case series of patients who had a mastectomy, after the procedure was early identified a chronic discomfort, which was then known as intercostobrachial nerve entrapment syndrome (5, 6). Post-mastectomy pain syndrome (PMPS) is the current name for this illness. According to the International Association for the Study of Pain, PMPS is today recognized as a persistent, neuropathic pain in the anterior surface of the chest axilla, shoulder, or upper arm that appears shortly after mastectomy or lumpectomy (6). Due to its potential multifactorial nature (7) and partial neuropathic origin (8), the reason for post-mastectomy chronic pain remains unknown. Numerous studies do not entirely support the hypothesis that surgical variables, such as the dissection and rebuilding of axillary lymph nodes, are significant risk factors for persistent pain (9, 10). Persistent pain following mastectomy has also been sporadically linked to adjuvant treatments like hormone therapy, chemotherapy, and radiation (11, 4). Individual differences in pain processing and results are significantly influenced by psychosocial factors, including anxiety, catastrophizing, and sleep disturbances (12, 13). Given that ongoing pain impairs alertness and, in turn, sleep quality, there is strong evidence linking chronic pain to sleep disorders (4).

Chronic pain and insomnia are therefore closely related, and long-term sleep deprivation further impairs mental and physical health, resulting in melancholy, chronic weariness, and anxiety in many individuals (12, 13). Immuno-neuro-endocrine diseases (14) have been linked to sleep disturbances, and these conditions are linked to the occurrence of chronic pain in general (15, 16).

The term “sleep quality” describes a complex process that includes both the depth and length of sleep (17). Insomnia, breathing disorders, central disorders of hypersomnolence, circadian rhythm sleep-wake disorders, sleep-related movement disorders, parasomnias, and other sleep disorders are examples of conditions that involve both subjective and objective symptoms (17).

Sleep quality and human physiological circadian rhythms are intimately related (18). There are noticeable diurnal cycles in the hormones cortisol and melatonin as well as core body temperature (19–21). The core body temperature rises during the day and falls at night. The hormones cortisol and melatonin are counter-regulatory, melatonin levels rise at night when cortisol levels fall to their lowest degree, and cortisol levels rise to their maximum in the morning when melatonin levels fall to their lowest degree. Sleep issues may result from abnormal cortisol and melatonin cycles (22, 23).

Poor sleep has been linked to risk-taking behavior, injuries, higher mortality rates, and a lower quality of life (24–27). Inadequate and poor-quality sleep is a high-risk factor for health outcomes like metabolic dysfunction, cardiovascular disease, and cognitive impairment.

Physical activity has been widely recognized as a key behavioral factor promoting psychological and physiological recovery after cancer treatment. Regular activity contributes to improved cardiorespiratory fitness, reduced fatigue and pain, enhancing quality of life in breast cancer survivors (28–31). As defined by the World Health Organization (WHO), physical activity is considered any bodily movement produced by skeletal muscles that requires energy expenditure. Physical activity is referred to all movements, including during leisure time, for transport to get to and from places, or daily activities. Both moderate- and vigorous-intensity physical activity improves mental health and reduces the risk of developing noncommunicable diseases such as heart disease, stroke, diabetes, and cancers, while exercise represents a planned and structured subset of physical activity aimed at improving physical fitness (32, 33). Importantly, exercise and habitual physical activity are also closely linked to the regulation of the sleep–wake cycle and neuroendocrine homeostasis. Several studies have shown that both structured physical exercise and habitual physical activity can help improve sleep quality by raising melatonin levels and modulating cortisol concentrations (34–36). Recent studies have highlighted the potential benefits of physical activity for breast cancer survivors, particularly in addressing post-mastectomy pain, insomnia, and sleep quality. Moderate-intensity aerobic activity and light resistance training have been associated with reductions in post-surgical pain and improvements in functional recovery (37, 38). Physical activity also improves sleep efficiency and reduces insomnia symptoms following breast surgery (39, 40).

It is emerging that type of exercise intervention substantially influences outcomes in cancer survivors. Combined aerobic and resistance training appear to provide the most consistent benefits across physical and psychosocial domains, including improvements in health-related quality of life, fatigue, and physical function (41, 42). Resistance training alone, has shown pronounced effects on muscle strength and body composition and may also reduce cancer-related pain (43). Aerobic exercise programs, particularly moderate-intensity walking or cycling, have been associated with better sleep quality and mood, while mind–body modalities such as Tai Chi and yoga can alleviate fatigue and anxiety and improve overall well-being (44, 45). However, evidence concerning neuroendocrine and inflammatory biomarkers (e.g., cortisol, melatonin, cytokines) remains mixed, likely due to small sample sizes, heterogeneous exercise protocols, and variable sampling methods (40).

By increasing melatonin hormone levels, decreasing estrogen production, and enhancing fat metabolism, physical activity has been demonstrated to lessen human cell deterioration or cancer susceptibility (46, 47). Furthermore, by modifying physiological variables, including DNA damage, cytokines, reactive oxygen species (ROS), and hormone levels, positive apoptotic regulation was noted in a variety of human cell types after engaging in moderate exercise training (48, 49). Because stress is a common barrier to falling and staying asleep, physical activity can help people fall asleep more quickly and improves quality of sleep. Exercise, also, physiologically modulates control of body temperature, which is essential for sleeping since a rise in body temperature during exercise promotes a subsequent reduction in body temperature 30–90 min after exercise, making it easier to fall asleep (50).

It has been observed that breast cancer patients are highly vulnerable to different sleep disturbances, and it seems that half of breast cancer survivors have sleep disorders (13). Sleep disorders represent a serious risk for breast cancer survivors, by causing severe physical and psychological consequences, worsening QOL, immunity, cognition, and mood in these patients (51).

On the light of the above considerations, the aim of the study was to investigate the relationship between physical activity, pain intensity, sleep quality, and neuroendocrine biomarkers (cortisol, ACTH, and melatonin) in women with persistent post-mastectomy pain.

## Materials and methods

### Study design

A prospective observational unicentric cohort study was conducted. The population of the study was represented by female patients who underwent unilateral or bilateral mastectomy due to the removal of stage II and III breast cancer, and who had not yet been subjected to chemotherapy, or radiation, aged 18 years or over. Pain evaluation for each participant in the study was assessed at 3 and 6 months after surgery through the verbal administration of the Numerical Rating Scale (NRS). Pittsburgh Quality of Sleep Index (PQSI) and Insomnia Severity Index (ISI) were used for sleep quality assessment. Physical activity was measured at 3 and 6 months after surgery with the International Physical Activity Questionnaire (IPAQ). At the same timepoints, the following blood biomarkers associated with sleep disorders were evaluated: cortisol, adrenocorticotropic hormone (ACTH), and melatonin.

### Participants

Enrollment was performed between April and October 2023 at the Breast Unit of San Vincenzo Hospital of Taormina in collaboration with the Azienda Ospedaliera Universitaria (AOU) Policlinico “G. Martino” of Messina, Italy. Inclusion criteria for patients were to be women aging over 18 years with a prior diagnosis of Phase II or III breast cancer who had undergone mastectomy for cancer removal within 3 months earlier. As exclusion criteria were considered: chemotherapy and radiation throughout the 3 or 6 months after surgery, anamnesis including other cancer types, immune system disorders (multiple sclerosis, HIV, lupus), and fresh flu symptoms (cough, fever). Women taking anxiolytic and/or antidepressant and anti-inflammatory drugs in the 15 days before recruitment were also excluded. Patients with a diagnosis before surgery of breast cancer at Stages 0 and I were not included because of the possible and frequent lack of pain. Women with cancer at Stage IV were not included in the study, as pain can derive from metastases. Women reporting pain before surgery and those affected by other types of tumors or other diseases characterized by chronic pain were also excluded. All patients were asked to sign the informed consent form to be included in the study. The study was approved by the Ethics Committee of AOU Policlinico “G. Martino”: Approval Number: Prot. 90-24, 16 July 2024, Board Name: Comitato Etico Interaziendale Messina. The trial was conducted according to the ethical principles of the Declaration of Helsinki, and Good Clinical Practice principles were adopted. To enroll subjects in the study, sample size was calculated using Clinicalc.com (https://clincalc.com/, accessed on 17 July 2024), (ClinicalTrials.gov Identifier: NCT06803563).

### Demographic and surgical variables

Demographic variables influencing pain-related conditions, including age, marriage, and school level, were considered. The Italian education system comprises primary (five years), secondary (three years), post-secondary (five years), and graduation phases (three-six years). Data about lymph nodes dissection were collected for each participant.

### Groups classification

Participants will first be classified according to the presence or absence of pain, based on the NRS. Within the pain group, participants are further stratified according to sleep disturbances, resulting in subgroups with and without sleep problems. Physical activity is then assessed within these sleep-based subgroups to explore its potential interactions with sleep quality. Biomarkers, however, are measured and analyzed across all groups, irrespective of pain or sleep status, to evaluate their associations with both pain and sleep disturbances. This hierarchical grouping allows for a structured analysis of primary and secondary factors while ensuring that all variables, including biomarkers, are consistently evaluated across the study population. All the data were collected 3 and 6 months after surgery.

### Numerical rating scale (NRS)

The Numeric Rating Scale (NRS), a validated tool for assessing pain intensity (52), was administered at 3 and 6 months post-surgery to classify participants according to the presence or absence of pain, as previously described (53).

The NRS is a 0–11 points scale with endpoints representing the extremes, meaning no pain (point 0) and the worst possible pain (point 10) (54, 55). The score allows the identification of three cutpoints to establish the degree of change representing clinical improvement. Three levels of pain severity were considered as follows: ratings of 1–4 correspond to mild pain, 5–6 to moderate pain, and 7–10 to severe pain (39). Participants were categorized into two groups according to the presence or absence of post-mastectomy pain (PMP), as assessed by the Numeric Rating Scale (NRS). Women reporting any pain intensity (NRS ≥5) persisting for at least 3 months after surgery were included in the PMP group, whereas those reporting no pain (NRS < 5) were classified as the non-PMP group.

### Pittsburgh sleep quality index (PSQI)

The PSQI is a self-report inventory assessing sleep duration, latency, and overall sleep quality over 1 month time interval. Each item is measured on a 0–3 interval scale. A score of 0 indicates no difficulty, while a score of 3, indicates severe difficulty. The global PSQI score is calculated by totalizing the seven component scores, providing an overall score ranging from 0 to 21, where lower scores indicate a healthier sleep quality. A score ≤ 5 generally reflects a good sleep quality while a score > 5 reflects a bad sleep quality (56).

### Insomnia severity index (ISI)

The ISI is a self-rated instrument with seven items that assess insomnia's nature, severity, and impact, rated on a 5-point [0 (no problem) – 4 (very severe problem for each item)]. Insomnia is graded as absent (score 0–7), mild (score 8–14), moderate (score15–21), and severe (score 22–28) (57).

### International physical activity questionnaire (IPAQ)

The International Physical Activity Questionnaire (IPAQ) was used to collect information about self-reported physical activity. It was administered 3 and 6 months after surgery, as previously described (52). This questionnaire measures the type and amount of physical activity. It assesses the number of days and quantity of time spent on physical activity of moderate or vigorous intensity, walking for at least 10 min during the last 7 days, and also comprises the time spent sitting during the last week. The IPAQ includes four physical activity levels (work-related activity, leisure-time activity, transport-related activity, and domestic activities), each with 3 degrees of intensities: walking, moderate, and vigorous. Whole weekly physical activity was evaluated by weighing time consumed in each activity intensity together with its calculated metabolic equivalent energy expenditure (Metabolic Equivalent of Task, MET). According to the answers, patients were classified into three categories: inactive, if presenting a METs less than 700, adequate active women presenting a METs value raging between 700 and 2519, and highly active if presenting METs > 3,000 (58).

### Haematological biomarkers associated with sleep disorders

Serum levels of cortisol, ACTH and melatonin were measured 3 and 6 months after surgical intervention, according to the protocol of ELISA kits. Blood samples were collected between 7 and 8 a.m. under fasting conditions. The following kits were used: ACTH (Novus biologicals, Novus Biologicals USA 10730 E. Briarwood Avenue Centennial, CO 80112, USA, NBP2-66401), cortisol (Novus biologicals NBP3-18003), melatonin (Invitrogen, Catalog # EEL056).

### Statistics

The Mann–Whitney *U*-test or Wilcoxon test was used to compare independent groups and paired data, respectively. Data are presented as mean ± standard deviation, and significance was set with a *p*-value < 0.05. Spearman's rank correlation was applied to explore the relationships between the biomarkers and the scores from the NRS, BDI, GAD-7, and IPAQ questionnaires. In addition, correlations were assessed both among the questionnaire scores themselves and among the biomarkers, in order to investigate potential interrelationships within and between these measures. According to the test, the correlation is considered “very weak” for values between 0.00 and 0.19, “weak” for values between 0.20 and 039, “moderate” for values between 0.40 and 0.59, “strong” for values between 0.60 and 079, and “very strong” for values between 0.80 and 1.0.

## Results

### Participants

Two hundred (200) female patients who underwent mastectomy were selected for the study, and one hundred and eighty (180) women were enrolled in the study according to inclusion and exclusion criteria. The mean age of the sample of patients recruited for the study was 50.34 ± 11.9 years (range 28–72 years, median age 53.5 years). The mean BMI was 21.59 ± 1.49. All the participants were Caucasian (Table 1).

**Table 1:** Age, education level, lymph nodes dissection, and marital status in PMP and non-PMP groups.

Group	Age (years)	Education level	Lymph nodes dissection	Marital status	NRS
Non-PMP (*n* = 94)	48.71 ± 11.66	Secondary = 10.33% Post-secondary = 30.81% Graduation = 11.66%	31.26%	41.94%	146 ± 1.51
PMP (*n* = 86)	50.66 ± 11.55	Secondary = 9.7% Post-secondary = 24.41% Graduation = 12.91%	34.78%	40.81%	5.93 ± 1.25*

PMP = post mastectomy pain group totalizing an NRS score ≥ 5, non-PMP group post-mastectomy pain group totalizing an.

NRS score < 5, NRS = Numerical Rating Scale * = *p* < 0.01 vs. non-PMP group.

### Numerical rating scale score (NRSs)

Clinical examination and estimation of NRS results collected 3 months after surgery showed that 52.2% (*n* = 94) did not report any significant pain (non-PMP group), while 47.7% (*n* = 86) of women recruited for the study manifested pain (post mastectomy pain, PMP). In consequence of this, participants were divided into two groups, the PMP group and non-PMP group.

In the group of PMP patients, the assessment of NRS performed either 3 or 6 months after surgery revealed a statistically significant higher degree in pain intensity in PMP patients compared with those of the non-PMP group. No intragroup differences at 3 months or 6 months were showed neither for PMP group or non-PMP group (Table 2).

**Table 2:** Evaluation of intensity of pain, melatonin, ACTH and cortisol in post-mastectomy pain (PMP) and non-post-mastectomy pain (non-PMP) groups at 3 and 6 months after surgery.

Outcome measures and biomarkers	Non-PMP *n* = 94		PMP *n* = 86	
	3 months	6 months	3 months	6 months
NRS score (intensity of pain)	1.44 ± 1.86	1.51 ± 1.77	6.02 ± 1.16*	5.90 ± 1.02*
Melatonin (pg/mL)	44.93 ± 4.69	45.29 ± 4.40	31.52 ± 7.44*	31.10 ± 6.91*
ACTH (pg/mL)	11.66 ± 2.14	12.10 ± 2.22	220.55 ± 305.73**	220.50 ± 304.19*
Cortisol (ng/mL)	6.10 ± 2.02	5.95 ± 2.10	60.58 ± 38.41*	60.27 ± 38.89*

PMP = post-mastectomy pain group totalizing an NRS score ≥ 5, non-PMP = post-mastectomy pain group totalizing an NRS score < 5, NRS = Numerical Rating Scale, ACTH = adrenocorticotropic hormone. Data are expressed as mean ± standard deviation. The Mann–Whitney *U*-test and Wilcoxon test were used to compare independent groups and paired data, respectively.

* = *p* < 0.01 vs. non-PMP group.

### Pittsburgh sleep quality index (PSQI) and insomnia severity index (ISI)

The Pittsburgh Sleep Quality Index (PSQI) and the Insomnia Severity Index (ISI) were used to evaluate sleep quality in women with Post-Mastectomy Pain Syndrome (PMP). Results revealed that 59.3% of these patients (*n* = 51) experienced poor sleep quality both at 3 and 6-months post-surgery, while 40.7% (*n* = 35) maintained healthy sleep quality (Table 2).

The subgroup with poor sleep was identified as the Sleep Disturbance PMP group (SD-PMP). In this group, assessments of PSQI and ISI conducted at 3 and 6 months demonstrated a statistically significant increase (*p* < 0.01) in scores at both the two timepoints, indicating in this group a worse quality of sleep compared to data of the non-SD-PMP group, evaluated with the two questionnaires (Table 2).

### Biomarkers related to sleep disorders

Cortisol, and ACTH levels were significantly increased in the PMP group compared to the non-PMP group, while melatonin was significantly reduce compared to non-PMP group. These biomarkers were significantly more elevated in the SD-PMP subgroup in comparison with the non-SD-PMP subgroup, either 3 or 6 months after surgery (Tables 2, 3). Melatonin levels were statistically significantly reduced in the SD-PMP subgroup compared to the non SD-PMP subgroup (Table 3).

**Table 3:** Evaluation of intensity of pain, and melatonin, ACTH and cortisol in SD-PM subgroup and non SD-PMP subgroup at 3 and 6 months after surgery.

Outcome measures and biomarkers	SD-PMP (*n* = 51)		non SD-PMP (*n* = 35)	
	3 months	6 months	3 months	6 months
PISQ score	12.02 ± 3.54*	14.5 ± 3.73*	1.85 ± 0.69	1.41 ± 0.62
ISI score	14.14 ± 3.7*	13.88 ± 3.75°*	2.34 ± 0.83	1.33 ± 0.52
NRS score	6.37 ± 1.34*	6.09 ± 1.23°*	5.51 ± 0.68	5.31 ± 0.47
Melatonin (pg/mL)	27.04 ± 6.47*	27.34 ± 6.36*	38.05 ± 1.67	36.58 ± 2.83
ACTH (pg/mL)	349.84 ± 382.43*	347.58 ± 381.52*	58.43 ± 4.11	58.73 ± 4.19
Cortisol (ng/mL)	79.87 ± 39.51*	79.17 ± 40.76*	32.48 ± 4.65	32.73 ± 4.58

SD-PMP = women reporting a PSQI ≥ 5 and/or ISI ≥ 7, non SD-PMP = women reporting a PSQI < 5 and/or ISI < 7, NRS = Numerical Rating Scale, PSQI = Pittsburg Sleep Quality Index, ISI = Insomnia Severity Index, ACTH = adrenocorticotropic hormone. Data are expressed as mean ± standard deviation. The Mann–Whitney *U*-test and Wilcoxon test were used to compare independent groups and paired data, respectively.

* = *p* < 0.01 vs. non SD-PMP.

° = *p* < 0.05 vs. 3 months.

### IPAQ score

Physical activity was evaluated with IPAQ within the group of 51 women of PMP group showing sleep disorders (SD-PMP). According to the IPAQ questionnaire, 23 patients (45%) of SD-PMP group have been categorized as inactive (<700 METs), and 28 (55%) as adequately active (>700 METs).

No active women (>2,510 METs) were identified in the SD-PMP group. SD-PMP inactive women showed a statistically significant increase in the intensity of pain (*p* < 0.01) and an increase in anxiety and depression scores (*p* < 0.01) compared to adequate active SD-PMP women, either 3 or 6 months after surgery (Table 4).

**Table 4:** Evaluation of physical activity by international physical activity questionnaire (IPAQ) in SD-PMP subgroup 3 and 6 months after surgery.

IPAQ score		
METs	SD-PMP (*n* = 51)	
Outcome measures and biomarkers	3 months after surgery	6 months after surgery
<700 (Inactive) (*N* = 20)	618.78 ± 66.23	625.17 ± 7
700–2,509 (Adequate active) (*N* = 24)	1,081.61 ± 246.89*	1,173.52 ± 213.13*°
>2,510 (Active) (*N* = 0)	N.D	N.D

SD-PMP = women reporting a PSQI ≥ 5 and/or ISI ≥ 7, non SD-PMP = women reporting a PSQI < 5 and/or ISI < 7, METs = Metabolic Equivalent of tasks, IPAQ = Intrernational Physical Activity Questionnaire.

* = *p* < 0.01 vs. inactive.

° = *p* < 0.01 vs. 3 months.

SD-PMP inactive women showed a statistically significant increase in the intensity of pain (*p* < 0.01) and an increase in PSQI and ISI scores (*p* < 0.01) compared to adequate active DA-PMP women, either 3 or 6 months after surgery (Table 5).

**Table 5:** Evaluation of the intensity of pain, and ACTH, cortisol and melatonin levels in the SD-PMP subgroup at 3 and 6 months after surgery, according to the physical activity score.

IPAQ score				
Outcome measures and biomarkers	Adequate Active SD-PMP (*n* = 28)		Inactive SD-PMP (*n* = 23)	
	3 months	6 months	3 months	6 months
PISQ score	9.18 ± 1.09*	8.60 ± 0.58*	15.48 ± 2.08	15.69 ± 2.20
ISI score	11.29 ± 2.27*	10.30 ± 1.79°*	17.61 ± 1.37	17.52 ± 1.23
NRS score	5.36 ± 0.55*	4.78 ± 0.67°*	7.61 ± 0.89	7.11 ± 0.95
Melatonin (pg/mL)	31.60 ± 5.14	33.68 ± 2.24	21.49 ± 2.02	21.18 ± 2.16
ACTH (pg/mL)	144.11 ± 54.06*	129.73 ± 48.05°*	560.29 ± 433.14	560.20 ± 429.24
Cortisol (ng/mL)	54.07 ± 11.65*	48.68 ± 10.26*	111.28 ± 38.77	111.58 ± 39.79

SD-PMP = women reporting a PSQI ≥ 5 and/or ISI ≥ 7, non SD-PMP = women reporting a PSQI < 5 and/or ISI < 7, NRS = Numerical Rating Scale, PSQI = Pittsburg Sleep Quality Index, ISI = Insomnia Severity Index, ACTH = adrenocorticotropic hormone. Data are expressed as mean ± standard deviation. The Mann–Whitney *U*-test and Wilcoxon test were used to compare independent groups and paired data, respectively.

* = *p* < 0.01 vs. non SD-PMP.

° = *p* < 0.05 vs. 3 months.

Cortisol, and ACTH levels were statistically significantly increased in inactive SD-PMP women compared to active SD-PMP women (Table 5), while melatonin levels were statistically significantly reduced in inactive SD-PMP women compared to active women of the same group.

Moreover, the SD-PMP active women showed, 6 months after surgery, a statistically significant reduction of ISI score and ACTH levels, compared to 3 months after surgery (Table 5).

### Spearman's correlation

Spearman's correlation was performed to analyze the relationship between scores obtained with questionnaires NRS, PSQI and ISI, and the biomarkers investigated (melatonin, cortisol, and ACTH).

**Figure 1:**
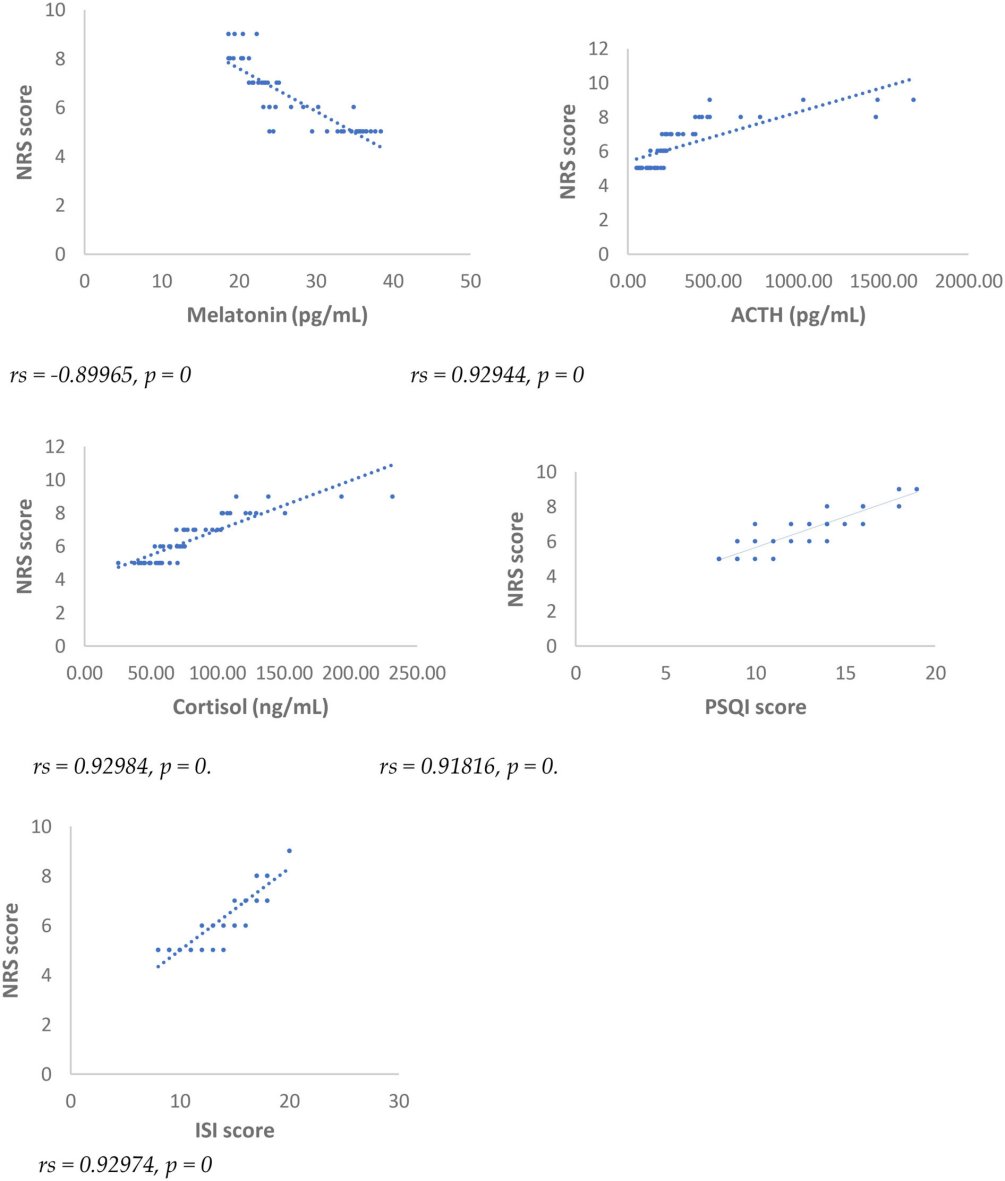
Spearman's correlation between numerical rating scale (NRS) score and melatonin, ACTH, cortisol, PSQI and ISI, in SD-PMP group, 3 months after surgery.

**Figure 2:**
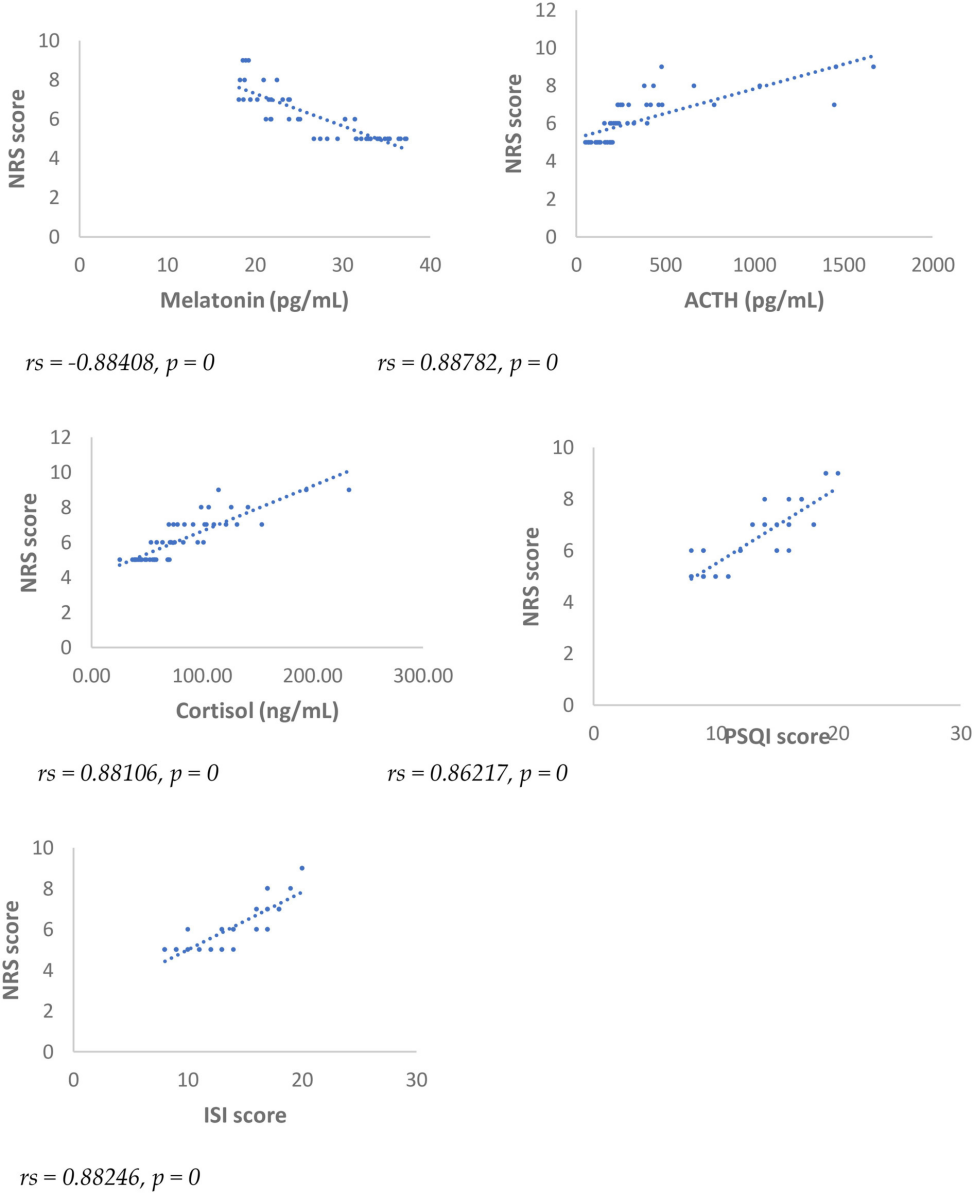
Spearman's correlation between numerical rating scale (NRS) score and melatonin, ACTH, cortisol, PSQI and ISI, in SD-PMP group, 6 months after surgery.

**Figure 3:**
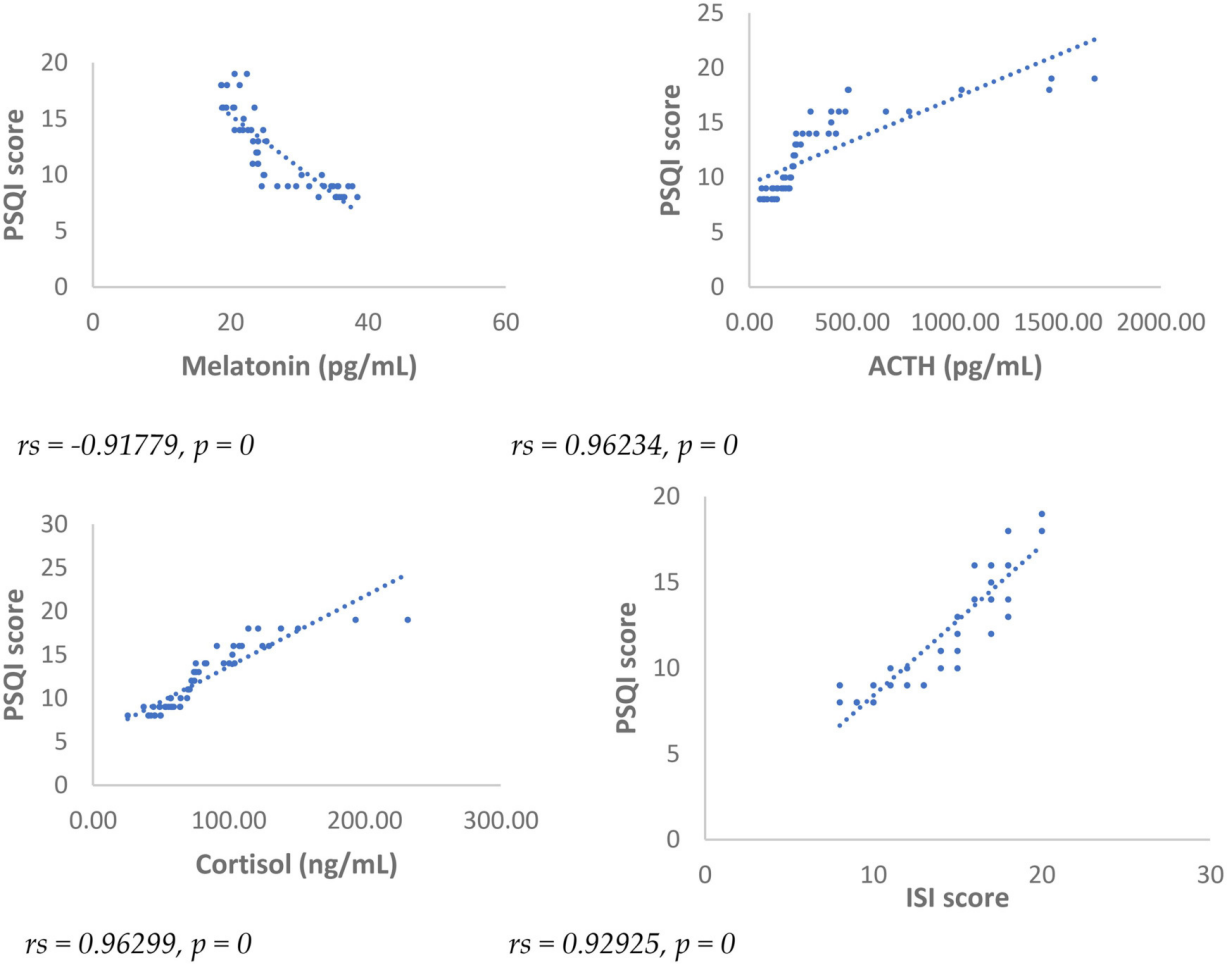
Spearman's correlation between pittsburg sleep quality Index (PSQI) score and melatonin, ACTH, cortisol, and ISI, in SD-PMP group, 3 months after surgery.

**Figure 4:**
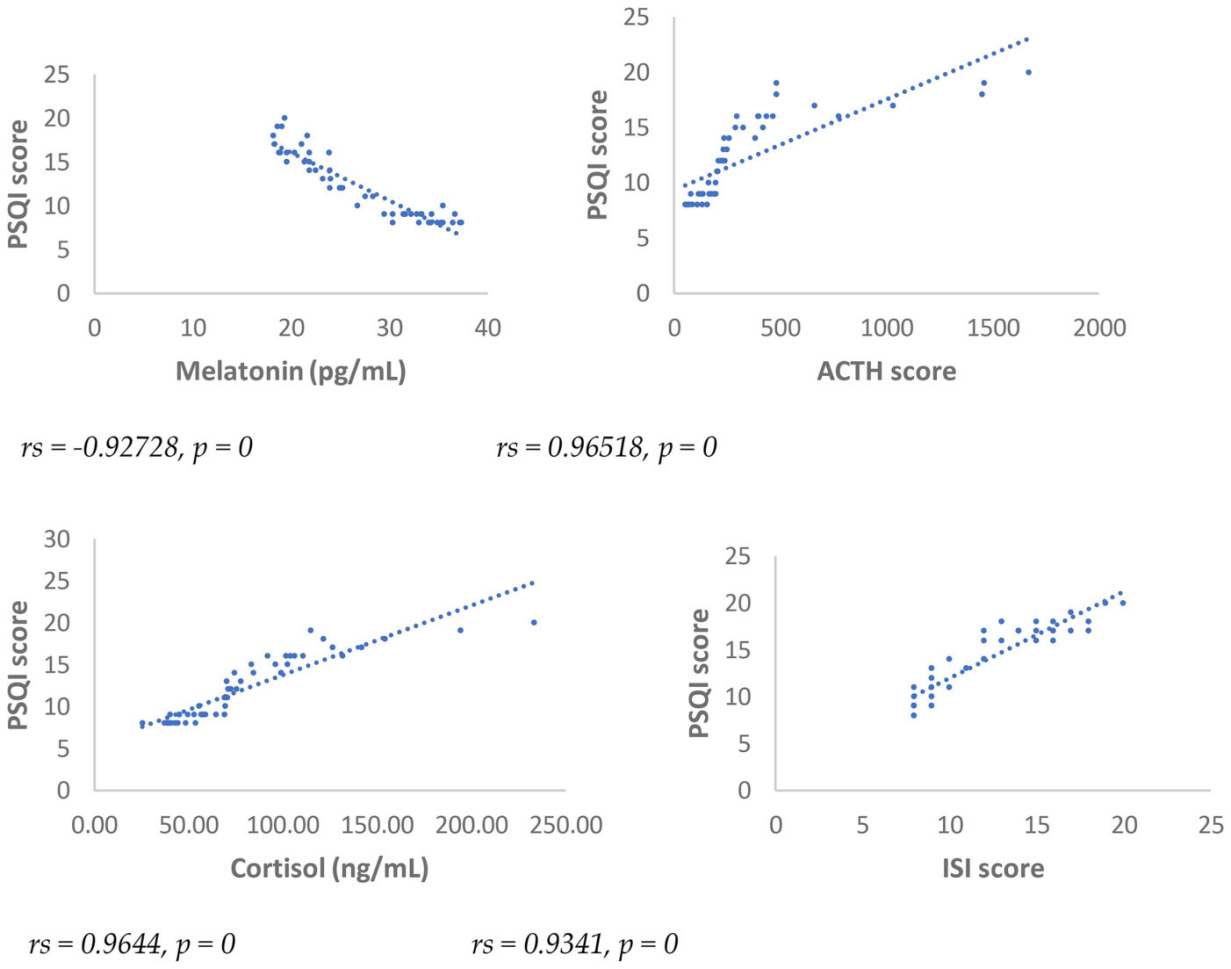
Spearman's correlation between pittsburg sleep quality Index (PSQI) score and melatonin, ACTH, cortisol, and ISI, in SD-PMP group, 6 months after surgery.

**Figure 5:**
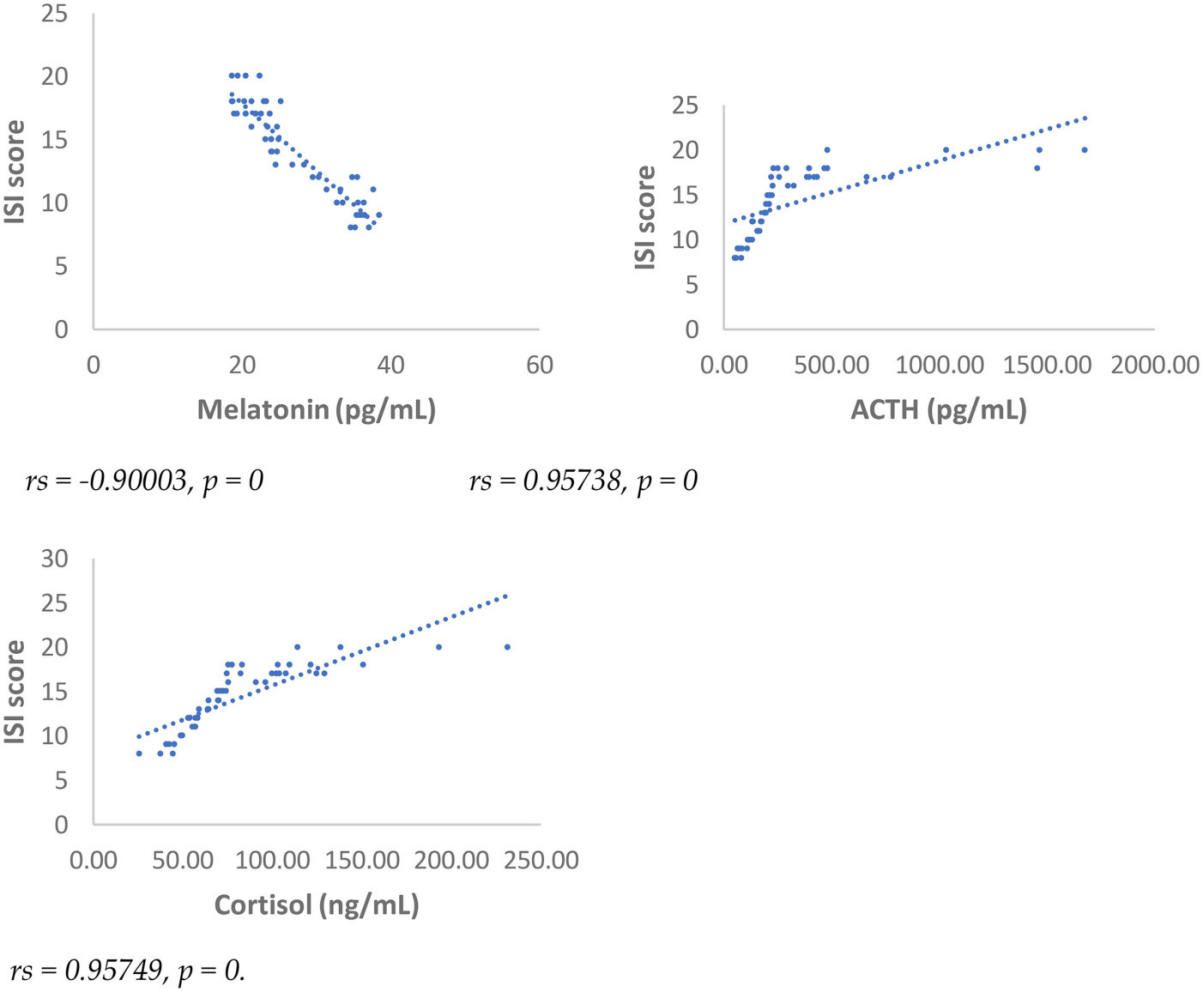
Spearman's correlation between sleep quality severity Index (ISI) score and melatonin, ACTH, cortisol, in SD-PMP group, 3 months after surgery.

**Figure 6:**
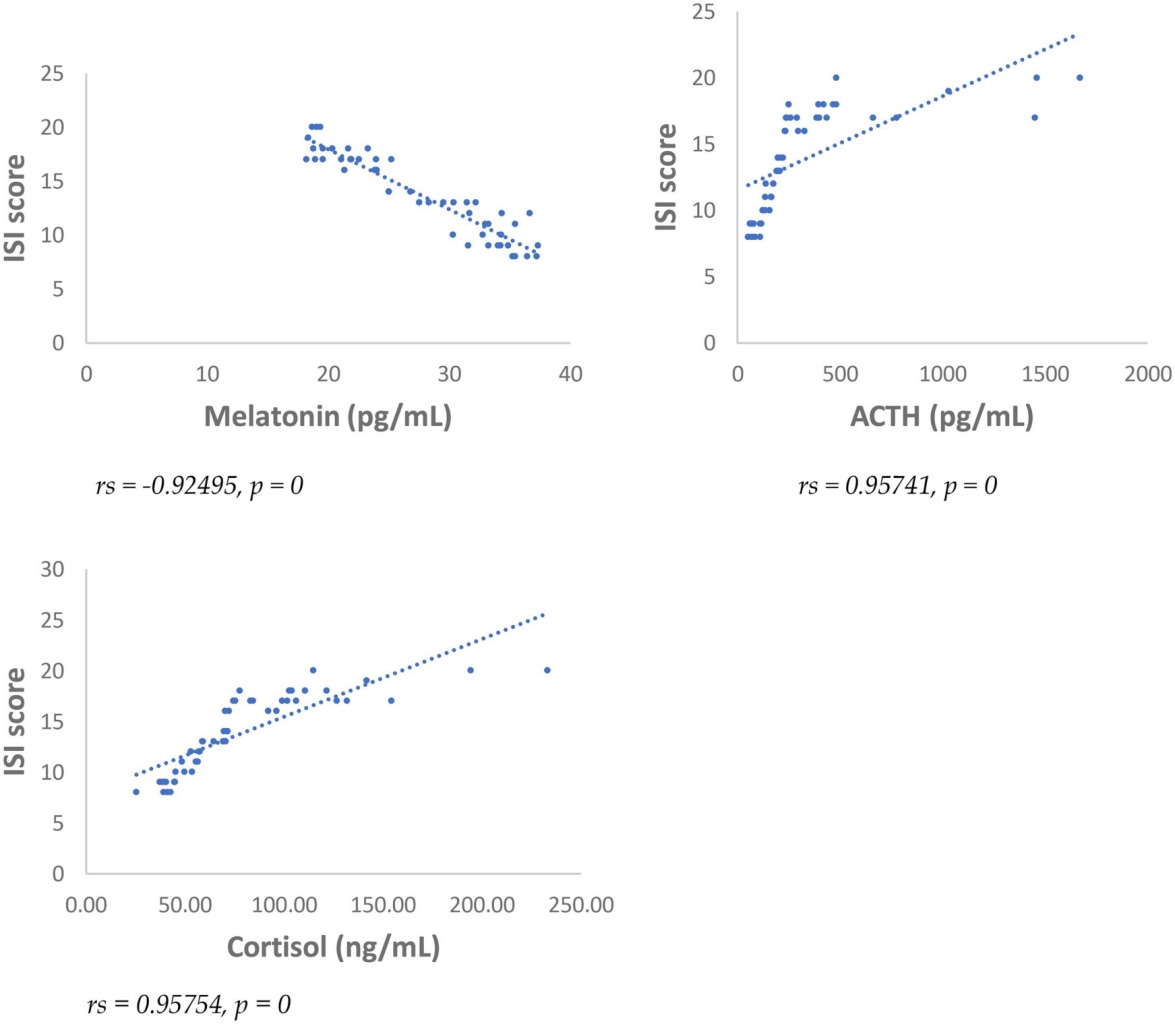
Spearman's correlation between sleep quality severity Index (ISI) score and melatonin, ACTH, cortisol, in SD-PMP group, 6 months after surgery.

Results showed at 3 and 6 months from surgery a statistically significant positive correlation (*p* < 0.001), between NRS, PSQI and ISI and cortisol and ACTH (Figures 1–6).

A statistically significant negative correlation (*p* < 0.001) has been observed between levels of melatonin and results deriving from NRS, PSQI and ISI, both 3 and 6 months after surgery, respectively (Figures 1–6).

The IPAQ score showed a statistically significant negative correlation (*p* < 0.001) with NRS, PSQI, ISI, cortisol and ACTH, 3 months after surgery and 6 months after surgery (Figuress 7,8).

**Figure 7:**
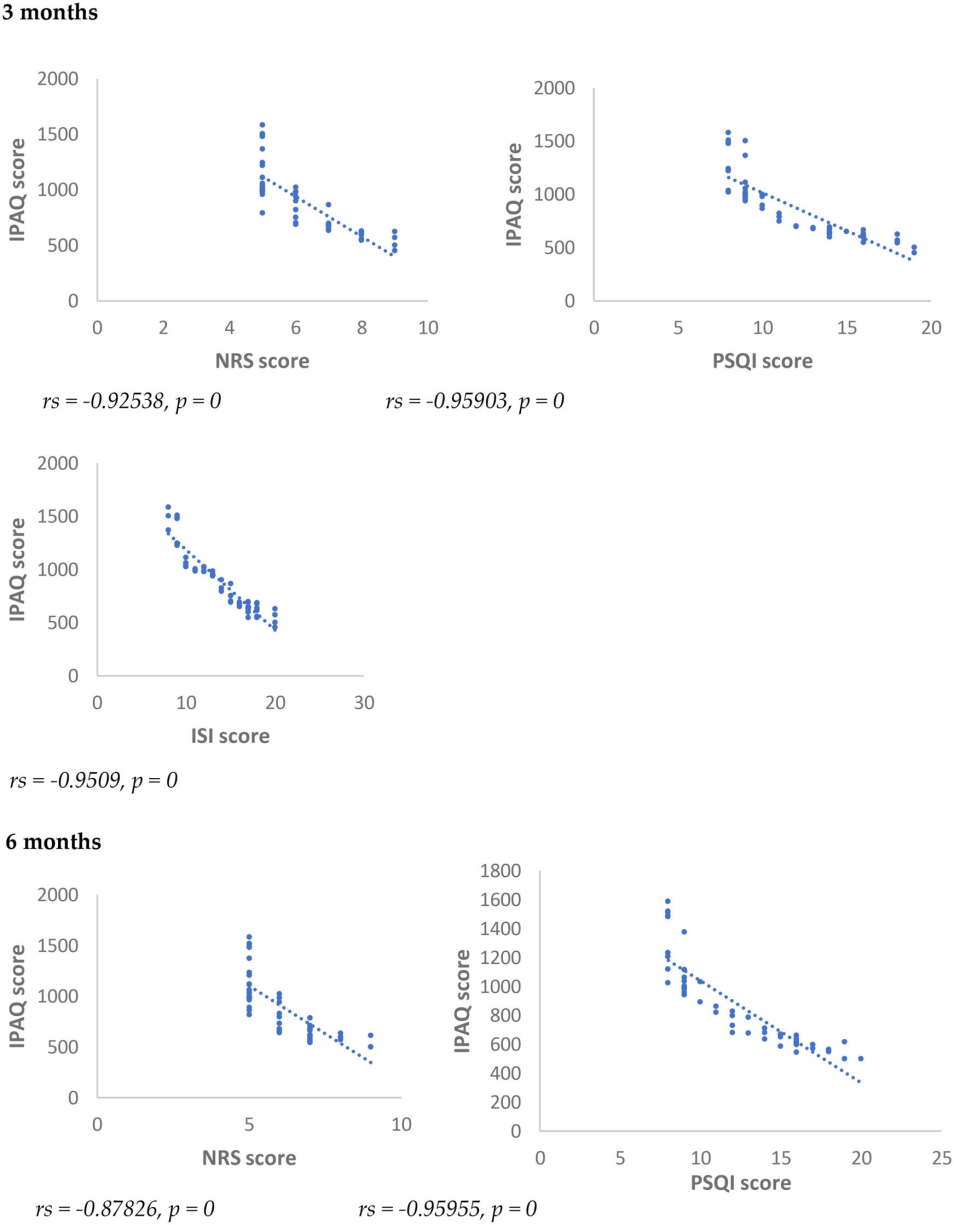
Spearman's correlation between international physical activity questionnaire (IPAQ)/NRS, IPAQ/PSQI and IPAQ/ISI score in SD-PMP group, 3 and 6 months after surgery.

**Figure 8:**
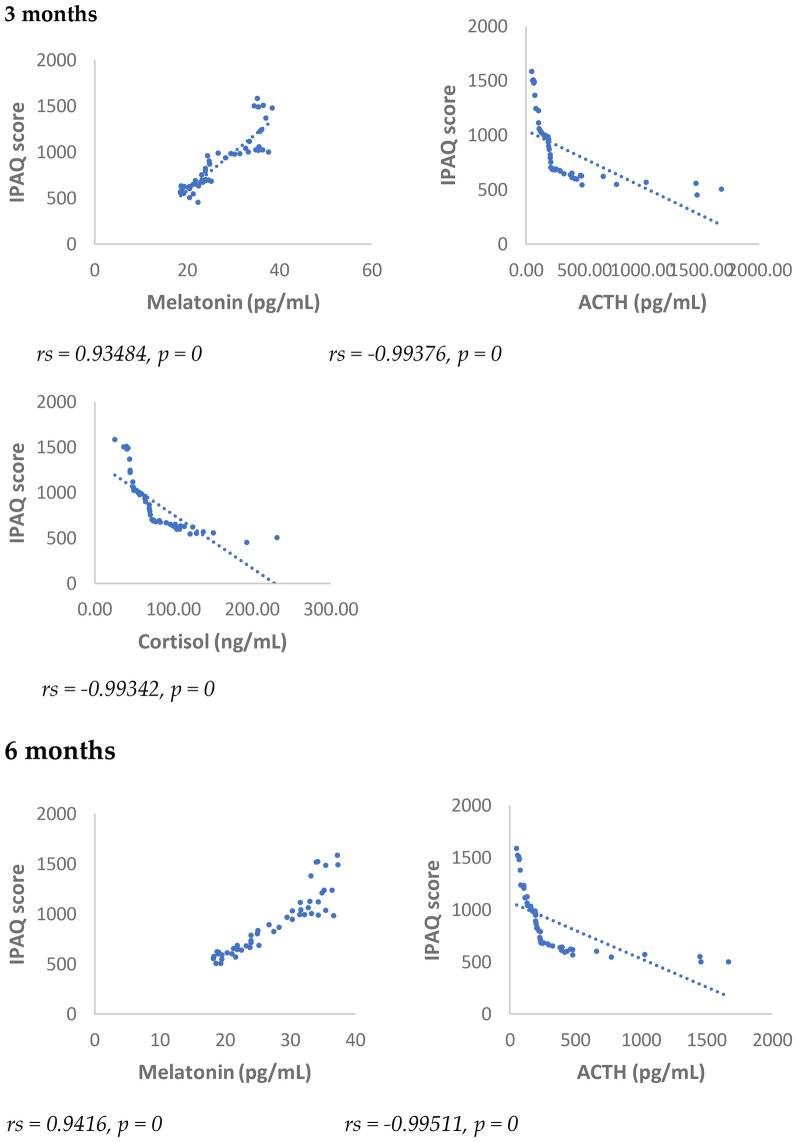
Spearman's correlation between international physical activity questionnaire (IPAQ) and melatonin, ACTH, and cortisol in SD-PMP group, 3 and 6 months after surgery.

On the contrary, the IPAQ score showed a statistically significant positive correlation (*p* < 0.001) with melatonin both at 3 and 6 months after surgery (Figure 8).

**Figure 9:**
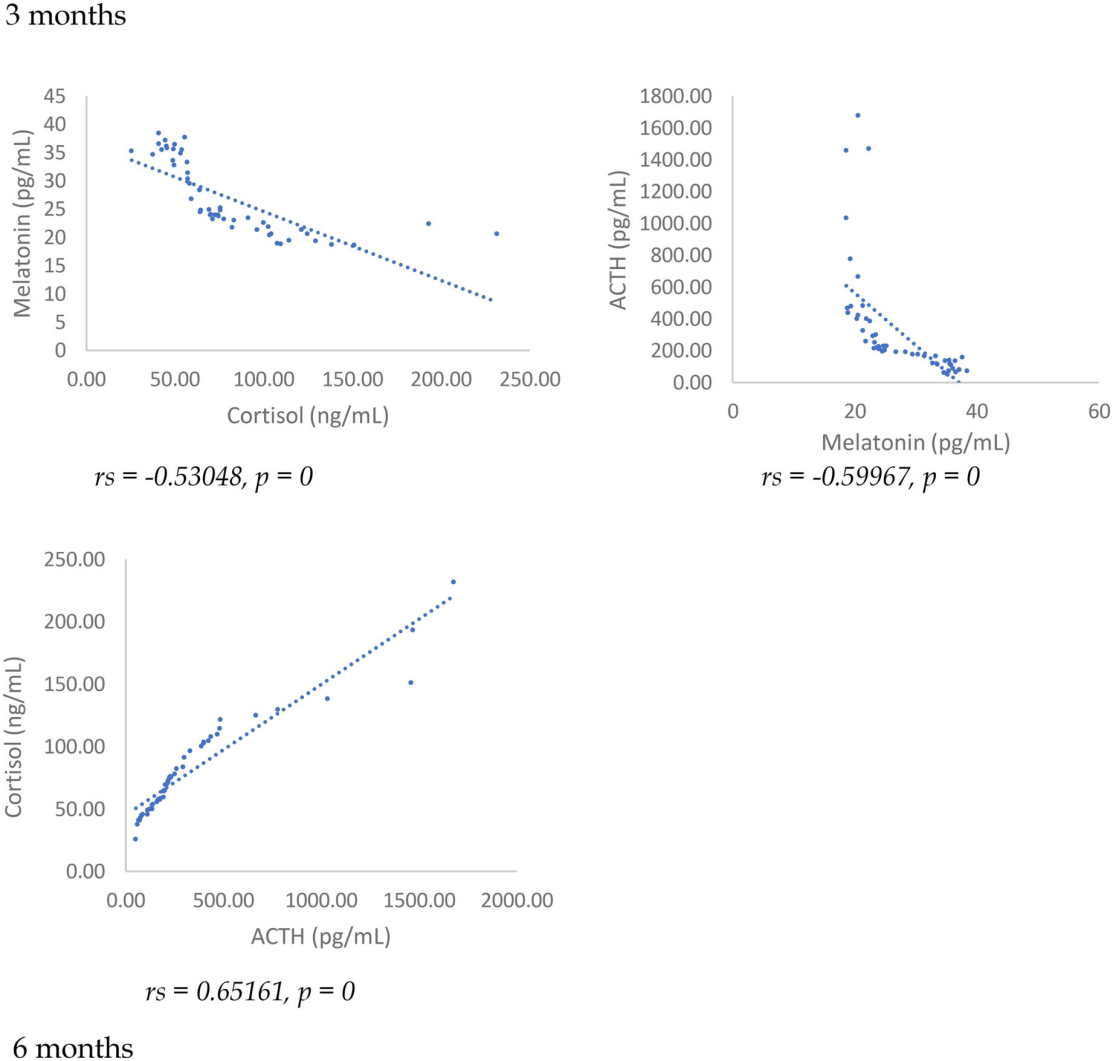
Spearman's correlation among melatonin, ACTH, and cortisol in SD-PMP group, 3 and 6 months after surgery.

Moreover, Speraman's correlations among biomarkers showed at 3 months, a significant positive association between cortisol and ACTH (*p* < 0.01), while melatonin was inversely and significantly correlated with both cortisol (*p* < 0.01) and ACTH (*p* < 0.01). At 6 months there was a significant negative relationship between melatonin and cortisol (*p* < 0.001), and melatonin and ACTH (*p* < 0.01). Between cortisol and ACTH, there was a positive and significant correlation (*p* < 0.01) (Figure 9).

## Discussion

The present study aimed to examine the associations between habitual physical activity, clinical symptoms, sleep quality, and neuroendocrine biomarkers in women after mastectomy. Our hypothesis was that higher levels of habitual physical activity would be associated with lower post-mastectomy pain, better subjective sleep quality, and a more balanced hormonal profile. The results supported this hypothesis, revealing meaningful links across behavioral, psychological, and biological domains (Table 1).

Our results, showed that the 47.7% of women who underwent breast cancer surgery experienced post mastectomy pain syndrome (PMPS).

Women experienced post mastectomy pain (PMP group) reported significantly higher NRS scores compared to non-PMP group, at both 3 and 6 months, confirming the chronic nature of this condition. No significant changes were observed within either group over time (3 vs. 6 months), suggesting that pain levels and related biomarkers remained relatively stable during the follow-up period.

Moreover an increase of ACTH and cortisol and a decrease of melatonin levels compared to non-PMP group has been shown.

The 59.3% of PMP women, reported sleep disturbances (SD). Also this group showed a statistically significant reduction aof melatonin level, and an increase of ACTH and cortisol, compared to PMP group not reporting sleep disturbance (non SD-PMP group).

However, in this study, 55% of SD-PMP patients were classified as physically inactive, while 65.5% were adequately active. None of the participants were categorized as fully active according to the International Physical Activity Questionnaire (IPAQ) standards. These results were consistent either at 3 or 6 months post-surgery.

Importantly, greater habitual physical activity, was inversely correlated with pain intensity. This association suggests that regular movement may exert protective or modulatory effects on chronic post-surgical pain. Possible mechanisms include the reduction of inflammatory mediators and the enhancement of descending pain inhibition pathways, both of which are influenced by regular activity (59). Previous studies in breast cancer survivors have also shown that higher daily activity levels are related to lower pain interference and improved functional recovery (30).

While our observational design prevents causal inference, the consistency of these patterns supports the potential role of physical activity as a behavioral regulator of pain persistence.

Participants reporting higher habitual activity displayed significantly better sleep quality (PSQI) and lower insomnia severity (ISI) scores. These results align with meta-analytic evidence indicating that regular physical activity improves sleep continuity and efficiency among breast cancer survivors (45).

The underlying mechanisms are likely multifactorial: increased physical activity may enhance circadian synchronization, reduce hyperarousal through HPA-axis modulation, and promote restorative slow-wave sleep. Notably, women with lower IPAQ scores exhibited both higher insomnia scores and flatter cortisol rhythms, indicating that reduced activity may contribute to circadian and hormonal dysregulation. These findings reinforce the idea that even habitual, non-programmed activity can sustain physiological homeostasis after oncologic surgery.

Moreover, the correlations observed between physical activity and hormonal measures provide further biological insight. At both 3 and 6 months, higher IPAQ scores were associated with lower morning cortisol and ACTH, and with higher nocturnal melatonin levels. This profile suggests a more adaptive regulation of the hypothalamic–pituitary–adrenal (HPA) axis and pineal function in physically active women. Such normalization may reflect a reduced allostatic load and improved circadian regulation, consistent with prior findings showing that exercise interventions can restore diurnal cortisol variability and melatonin amplitude (40, 44). As expected based on their physiological interactions, cortisol and ACTH showed a significant positive correlation, at 3 months after surgery, reflecting the well-established stimulatory role of ACTH on adrenal cortisol secretion. In contrast, melatonin was inversely but not significantly correlated with both cortisol and ACTH, which is consistent with the opposing circadian profiles of these hormones. At 6 months theserelstionship remained stable over time, with melatonin continuing to correlate negatively with cortisol and ACTH, and a persistent positive correlation between cortisol and ACTH. Such consistency suggests a robust and enduring regulatory interplay among these biomarkers. Mechanistically, melatonin's analgesic and anti-inflammatory properties may contribute to the concomitant improvements in pain and sleep.

Interestingly, the relationships between biomarkers and clinical outcomes were not strictly linear: scatterplots suggested asymptotic behavior, particularly between cortisol and pain or sleep indices, implying a possible saturation effect of the physiological stress response. This indicates that beyond a certain hormonal threshold, additional changes in cortisol may not translate into proportional symptom differences. Future analyses using non-linear modeling (e.g., generalized additive models or segmented regression) could clarify these dose–response relationships.

When considered together, our findings point to a coherent physiological pattern: habitual physical activity appears to buffer the negative effects of cancer treatment on both neuroendocrine regulation and behavioral outcomes. By stabilizing HPA axis dynamics and reinforcing circadian melatonin secretion, regular activity may indirectly improve both sleep and pain perception. The convergence between subjective (questionnaires) and objective (hormonal) indicators strengthens the interpretation that habitual movement acts as a low-intensity, self-regulating mechanism to support recovery and psychological well-being after mastectomy.

Despite the general consistency of our findings, inter-individual variability in the magnitude of benefit was evident. Genetic differences within HPA-axis and inflammatory pathways could partly explain this variability. NR3C1 variants and epigenetic modifications have been linked to altered glucocorticoid receptor expression and cortisol sensitivity (60, 61), IL6 and TNF-α polymorphisms have been shown to modify the acute and chronic cytokine response to exercise (62, 63), and the BDNF Val66Met allele attenuates exercise-induced increases in BDNF and related neuroplastic changes (64, 65). Recent genome-wide interaction studies also suggest that genetic variation can modify the relationship between habitual physical activity and biological responses (66–68). Integrating genetic profiling in future studies could thus help identify “responders” to physical activity and refine individualized rehabilitation approaches for breast cancer survivors.

Key strengths of this study include the longitudinal design, integration of subjective questionnaires and objective biomarkers, and focus on habitual activity rather than structured exercise interventions. This design enhances ecological validity and highlights the importance of everyday movement during recovery. However, several limitations should be acknowledged. The use of self-reported IPAQ data does not allow precise differentiation by exercise type, timing, or intensity. Moreover, pre-surgical pain measures were unavailable, and the relatively small sample size limited statistical power for subgroup analyses. Nonetheless, the consistent associations across pain, sleep, and hormonal variables provide convergent support for the role of habitual activity in recovery.

## Conclusions

Overall, our results suggest that higher levels of habitual physical activity are associated with lower pain, improved sleep quality, and more adaptive neuroendocrine regulation in women after mastectomy. These findings emphasize the potential of daily physical activity as a simple, low-cost, and holistic strategy to mitigate post-mastectomy symptoms and support endocrine balance. Future randomized studies should directly compare different activity modalities (aerobic, resistance, mind–body) and integrate objective activity monitoring with standardized biomarker sampling and genetic profiling to advance personalized physical rehabilitation strategies in cancer survivorship care.

## Data Availability

The raw data supporting the conclusions of this article will be made available by the authors, without undue reservation.
